# Diseases of the Antrum or Maxillary Sinus

**Published:** 1859-06

**Authors:** F. D. Thurmond

**Affiliations:** Dentist, Atlanta, Georgia


### ARTICLE IV.

*Diseases of the Antrum or Maxillary Sinus.* By F. D. THURMOND, Dentist, Atlanta, Georgia.

[CONTINUED FROM PAGE 480, APRIL NUMBER.]

*Inflammation of its lining membrane.*—Situated as this cavity is, it would but seldom be affected with inflammation were it not for local irritants in the form of diseased teeth, &c.

As the roots of the molar teeth very nearly, and occasionally do, penetrate this cavity, it is plain to be seen that when, in a diseased condition, they would be liable to excite inflammation in this delicate tissue. If the patient's general health is good, the inflammatory action from such local causes seldom prove serious, and immediately subsides upon the removal of the cause. In fact, it often subsides for a time, whilst the cause remains. Many of the cases which are thought to be neuralgia, or rheumatic affections, of the face, are nothing more or less than inflammation of this membrane.

When inflamed, this membrane secretes a much larger quantity of fluid than when in a healthy state. This fluid soon becomes vitiated, and continues to accumulate until the cavity is full, unless the inflammation previously terminates. When full the matter escapes through the nose, and may be detected at any time (unless the natural openings have become closed, by turning the head to one side, when the vitiated and offensive mucus immediately oozes out at the nose. This symptom attends other diseases of this cavity, also—such as ulceration of the lining membrane, &c.—Though the character of the matter discharged is somewhat different. Occasionally inflammation of this membrane terminates in suppuration, and the discharge is of course in the form of pus. Inflammation of this cavity frequently assumes a chronic form, and is sometimes kept up for a great while with but little inconvenience to the patient, save occasional returns of slight pains, and a vitiated and very offensive condition of the secretions, which the patient soon becomes so much accustomed to, as not notice it; though his friends, if he has any true ones, are apt to call his attention to it.

This state of things is frequently allowed to continue until the increasing morbid effects involves the bony walls of the superior maxillary.

In the treatment of this disease antiphlogistics, purgatives, fumigations, &c., often become necessary. But the most important point is to examine for, and remove the cause, which, I think, in ninety cases out of a hundred, will be found in the form of diseased teeth and roots of teeth.

[TO BE CONTINUED.]

